# Gestational Age of Delivery in Pregnancies Complicated by Diabetes

**DOI:** 10.31486/toj.20.0019

**Published:** 2020

**Authors:** Lorie M. Harper, Alan T. N. Tita, Joseph R. Biggio, Jen Jen Chang

**Affiliations:** ^1^ Department of Obstetrics and Gynecology, Center for Women's Reproductive Health, The University of Alabama at Birmingham, Birmingham, AL; ^2^ System Co-Chair of Women's Services, Ochsner Clinic Foundation, New Orleans, LA and Clinical Professor of Obstetrics and Gynecology, The University of Queensland Faculty of Medicine, Ochsner Clinical School, New Orleans, LA; ^3^ Department of Epidemiology, Saint Louis University, St Louis, MO

**Keywords:** *Diabetes mellitus–type 1*, *diabetes mellitus–type 2*, *gestational age*, *infant–small for gestational age*, *perinatal death*, *pregnancy in diabetics*, *stillbirth*

## Abstract

**Background:** The recommended gestational age to deliver pregnancies complicated by diabetes ranges from 34 to 39 weeks of gestation. The objective of this study was to determine the optimal gestational age for delivery of patients with diabetes to minimize perinatal death.

**Methods:** We extracted a population-based cohort of singleton, nonanomalous infants of diabetic pregnancies from the Missouri birth registry for the period January 1, 1989 to December 31, 2005 and compared perinatal outcomes of planned deliveries at 37, 38, 39, and 40 weeks to expectant management. Planned deliveries were identified by induction or cesarean delivery without documented medical or obstetric indications. The primary outcome was perinatal death, defined as stillbirth or neonatal death within 28 days of birth. Secondary outcomes were independent stillbirth, independent neonatal death, and a composite adverse neonatal event of assisted ventilation >30 minutes, birth injury, seizures, or 5-minute Apgar score ≤3. Groups were compared using *t* test and chi-square as appropriate.

**Results:** In 4,905 diabetic pregnancies reaching 37 weeks, 1,012 (20.6%) patients were insulin dependent. Overall, the risk of perinatal death at any gestational age examined was low (3/1,000 births or lower), as was the risk of the adverse perinatal outcome (<2%). When only patients who were insulin dependent were included in the analysis, the risk of perinatal death at any gestational age remained low at 6 per 1,000 births or fewer.

**Conclusion:** Delivery as early as 37 weeks is reasonable for women who have diabetes, although the absolute risk of perinatal death is low at 37 to 39 weeks.

## INTRODUCTION

Insulin, home blood glucose monitoring, antenatal testing, and oral hypoglycemic agents have revolutionized the management of diabetes during pregnancy,^1^ leading to drastic reductions in maternal and infant mortality attributable to diabetic complications. In 1925, the perinatal mortality rate of infants born to mothers with diabetes was 40/1,000 births, compared to a rate of approximately 5/1,000 births in 2000.^1^ Despite these improvements, the risk of stillbirth remains 3- to 4-fold higher in patients with pregnancies complicated by diabetes compared to patients without diabetes.^2^

At term (37 weeks and beyond), the risk of stillbirth during ongoing pregnancy must be balanced against the risk of neonatal death and neonatal morbidities, particularly neonatal respiratory distress. The American College of Obstetricians and Gynecologists (ACOG), the Society for Maternal-Fetal Medicine (SMFM), and the Eunice Kennedy Shriver National Institute of Child Health and Human Development (NICHD) provide a broad range of gestational ages at which delivering pregnancies complicated by diabetes may be appropriate—from 34 to 39 weeks of gestation—although delivery at 39 weeks is recommended by all in the setting of well-controlled diabetes without vascular disease, and the ACOG in 2019 recommended a range of 36 to 39 weeks.^3,4,5^ One reason for this broad range is the lack of data in a modern cohort of patients with diabetes for whom home blood glucose monitoring and antenatal testing are the standard of care. Additionally, pregnancies complicated by diabetes have been excluded from studies reporting adverse infant outcomes with early-term birth,^6,7^ and many physicians are concerned about the risk of stillbirth in diabetic pregnancies beyond 37 weeks.^4,5^ We therefore sought to determine the gestational age of delivery associated with the lowest risk of perinatal death in pregnancies complicated by diabetes.

## METHODS

This population-based, retrospective cohort study is based on the Missouri linked birth certificate and fetal and infant death certificate data. The database includes parental demographic information, medical and obstetric characteristics and complications, and neonatal status at birth for each birth that occurred in the state. The study was exempt from institutional board review as it used a deidentified linked birth and death certificate registry.

Our study sample consists of all women with pregnancies complicated by diabetes who delivered singleton births between 37^0/7^ and 41^6/7^ weeks of gestation in Missouri between January 1, 1989 and December 31, 2005. We selected this gestational age range because it corresponds to early-term birth, term birth, and late-term birth. In the Missouri birth certificate data, diabetes is coded as either insulin dependent or not insulin dependent. Consequently, distinctions between gestational and pregestational diabetes could not be made. If a woman had multiple diabetic pregnancies during the time frame, only the first qualifying pregnancy was included. We based gestational age on the birth certificate variable “clinical estimate of gestation” because it more accurately reflects gestational age at delivery than length of pregnancy calculated using the last menstrual period.^8^ Pregnancies complicated by major fetal anomalies and multifetal gestation were excluded as these conditions would impact the outcome of interest.

The exposure of interest for this study was the planned timing of delivery. Planned deliveries were identified by documentation of either induction or cesarean delivery without documented medical or obstetric indications for delivery or spontaneous labor. Induction and cesarean delivery were abstracted from the dataset using the obstetric procedure codes for induction and the method of delivery. Indications for delivery were determined from the labor complication codes and maternal medical risk factor codes. Deliveries that were considered indicated were excluded from the analysis and included stillbirth, preeclampsia/eclampsia, premature rupture of membranes, and hydramnios. Exclusion of these indications for delivery was intended to identify women who were delivered either electively or for the sole indication of diabetes. Deliveries that were not coded as being induced by the obstetric procedure codes were considered to be spontaneous labor and excluded from analysis with the indicated deliveries.

We compared patients with a planned delivery at 37, 38, 39, and 40 weeks to patients with expectant management beyond each of those gestational age periods (ie, planned delivery at 37^0/7^ to 37^6/7^ compared to expectant management at ≥38^0/7^ weeks). Patients included in the expectant management group were women with spontaneous labor during the gestational age range of interest, a medical/obstetric indication for delivery during the gestational age range of interest (eg, induction or cesarean indicated by stillbirth, preeclampsia/eclampsia, premature rupture of membranes, or hydramnios), and patients who delivered beyond the gestational age range of interest for any indication.

The primary outcome of interest was perinatal death, defined as stillbirth or neonatal death within 28 days of birth. Perinatal death was chosen as the primary outcome because the ultimate goal is the survival of neonates, not just prevention of stillbirth. Secondary outcomes were stillbirth and neonatal death, assessed independently. Additionally, we considered a composite adverse neonatal outcome of assisted ventilation >30 minutes, birth injury, seizures, or 5-minute Apgar score ≤3. To be considered to have experienced the composite adverse outcome, a patient must have experienced one or more of the components.

### Statistical Analysis

Descriptive statistics are used to summarize sample characteristics and outcomes of interest according to the planned timing of delivery at different gestational ages, with chi-square test used for categorical variables. In a subanalysis, we restricted the analytical sample to insulin-dependent patients only to determine the association between the planned timing of delivery and outcomes of interest by gestational age. All tests were 2-tailed, and *P*<0.05 was considered significant. All statistical analyses were performed using Stata, release 13 (StataCorp LP).

## RESULTS

Of 979,849 women with singleton pregnancies in the Missouri birth cohort, 4,905 women with diabetes who reached 37 weeks were identified. Of these, 1,012 (20.6%) were insulin dependent and 3,893 (79.4%) were non–insulin dependent. The Figure demonstrates the inclusion of subjects in each comparison group.

**Figure.**
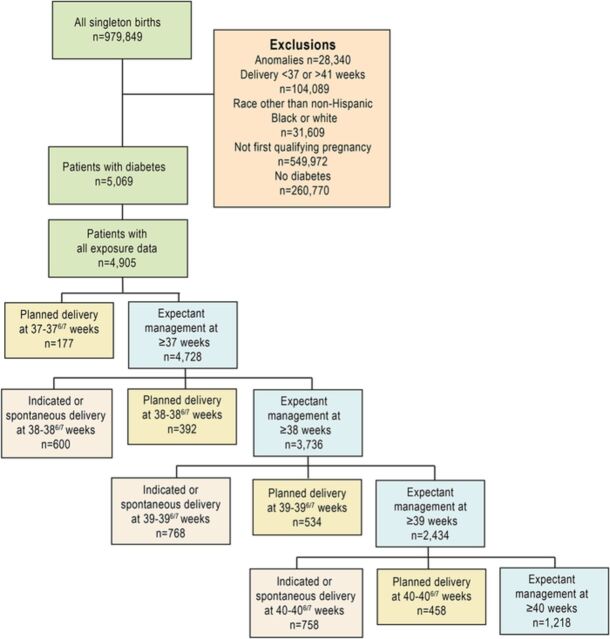
**Flowchart detailing the inclusion of subjects in each comparison group.**

### 37 Weeks

Between 37^0/7^ and 37^6/7^ weeks, 177 (3.6%) women underwent a planned delivery (Table 1). Compared to women expectantly managed beyond 37 weeks, women undergoing planned delivery at 37 weeks were less likely to be nulliparous (81.8% vs 89.6%, *P*<0.01), more likely to be obese (42.1% vs 32.2%, *P*<0.01), and more likely to receive adequate plus prenatal care (defined as ≥110% of expected visits,^9^ 75.0% vs 43.3%, *P*<0.01).

**Table 1.** Baseline Characteristics of Cohort (n=4,905)

	Gestational Age of 37^0/7^-37^6/7^ Weeks			Gestational Age of 38^0/7^-38^6/7^ Weeks			Gestational Age of 39^0/7^-39^6/7^ Weeks			Gestational Age of 40^0/7^-40^6/7^ Weeks		
Variable	Planned Delivery, n=177	Expectant Management, n=4,728	*P*	Planned Delivery, n=392	Expectant Management, n=3,736	*P*	Planned Delivery, n=534	Expectant Management, n=2,434	*P*	Planned Delivery, n=458	Expectant Management, n=1,218	*P*
Age, years			0.36			0.21			0.23			0.06
≤17	3 (1.7)	170 (3.6)		8 (2.0)	135 (3.6)		23 (4.3)	86 (3.5)		13 (2.8)	40 (3.3)	
18-25	76 (42.9)	2,033 (43.0)		160 (40.8)	1,617 (43.3)		206 (38.6)	1,053 (43.3)		179 (39.1)	558 (45.8)	
26-35	87 (49.2)	2,322 (49.1)		203 (51.8)	1,822 (48.8)		279 (52.2)	1,190 (48.9)		241 (52.6)	572 (47.0)	
≥36	11 (6.2)	203 (4.3)		21 (5.4)	162 (4.3)		26 (4.9)	105 (4.3)		25 (5.5)	48 (3.9)	
Race			0.94			0.24			0.93			0.09
White	158 (89.3)	4,212 (89.1)		358 (91.3)	3,340 (89.4)		482 (90.3)	2,194 (90.1)		424 (92.6)	1,094 (89.8)	
Black	19 (10.7)	516 (10.9)		34 (8.7)	396 (10.6)		52 (9.7)	240 (9.9)		34 (7.4)	124 (10.2)	
Nulliparous	144/176 (81.8)	4,224/4,713 (89.6)	<0.01	331/390 (84.9)	3,362/3,724 (90.3)	<0.01	472/532 (88.7)	2,196/2,427 (90.5)	0.22	410/455 (90.1)	1,096/1,215 (90.2)	0.95
WIC program	68 (38.4)	1,528 (32.3)	0.09	127 (32.4)	1,204 (32.2)	0.98	161/533 (30.2)	799/2,423 (33.0)	0.22	113/455 (24.8)	433/1,214 (35.7)	<0.01
Married	38 (21.5)	1,122 (23.7)	0.49	90 (23.0)	877 (23.5)	0.81	101 (18.9)	571 (23.5)	0.02	89 (19.4)	300 (24.6)	0.03
Body mass index	n=171	n=4,549	<0.01	n=378	n=3,589	<0.01	n=518	n=2,331	<0.01	n=440	n=1,168	0.12
Underweight	7 (4.1)	172 (3.8)		6 (1.6)	136 (3.8)		21 (4.1)	85 (3.7)		10 (2.3)	52 (4.5)	
Normal weight	49 (28.7)	1,858 (40.8)		130 (34.4)	1,473 (41.0)		187 (36.1)	965 (41.4)		169 (38.4)	476 (40.8)	
Overweight	43 (25.2)	1,055 (23.2)		97 (25.7)	835 (23.3)		108 (20.9)	551 (23.6)		110 (25.0)	283 (24.2)	
Obese	72 (42.1)	1,464 (32.2)		145 (38.4)	1,145 (31.9)		202 (39.0)	730 (31.3)		151 (34.3)	357 (30.6)	
Gestational weight gain, lb, mean ± SD	30.9 ± 14.5(n=170)	30.4 ± 15.0(n=4,561)	0.68	28.3 ± 15.5(n=379)	30.6 ± 15.0(n=3,601)	<0.01	30.0 ± 15.7(n=521)	30.5 ± 14.8(n=2,344)	0.49	30.3 ± 15.4	31.0 ± 14.8	0.42
Tobacco use	21/176 (11.9)	749/4,706 (15.9)	0.16	60 (15.3)	588 (15.7)	0.81	65/531 (12.2)	412/2,423 (17.0)	<0.01	70/454 (15.4)	207/1,214 (17.1)	0.43
Chronic medical illness	8 (4.5)	176 (3.7)	0.58	19 (4.9)	131 (3.5)	0.18	21 (3.9)	83 (3.4)	0.55	18 (3.9)	35 (2.9)	0.27
Prenatal care	n=172	n=4,575	<0.01	n=379	n=3,615	<0.01	n=520	n=2,346	<0.01	n=435	n=1,180	<0.01
Inadequate	9 (5.2)	251 (5.5)		14 (3.7)	199 (5.5)		26 (5.0)	137 (5.8)		20 (4.6)	76 (6.4)	
Intermediate adequate	5 (2.9)	340 (7.4)		6 (1.6)	320 (8.8)		14 (2.7)	274 (11.7)		35 (8.1)	178 (15.1)	
Adequate	29 (16.9)	2,005 (43.8)		146 (38.5)	1,688 (46.7)		211 (40.6)	1,179 (50.3)		205 (47.1)	646 (54.8)	
Adequate plus^a^	129 (75.0)	1,979 (43.3)		213 (56.2)	1,408 (39.0)		269 (51.7)	756 (32.2)		175 (40.2)	280 (23.7)	

Notes: Data are presented as n (%) unless otherwise noted. For variables with missing data, the number of available data points is shown.

^a^Adequate plus prenatal care is defined as ≥110% of expected visits.^9^

WIC, Women-Infants-Children.

The primary outcome of perinatal death occurred in no patients in the planned delivery and in 14 patients (0.3%) of the expectantly managed group (*P*=0.47, Table 2). Of these 14 perinatal deaths in the expectantly managed group, 8 deaths were stillbirths and 6 were neonatal deaths. The composite adverse neonatal event occurred in 3 (1.7%) planned deliveries between 37^0/7^ and 37^6/7^ weeks and in 68 (1.4%) of those expectantly managed to ≥38 weeks (*P*=0.78).

**Table 2.** Outcomes of Planned Delivery vs Expectant Management for All Patients (n=4,905)

	Gestational Age of 37^0/7^-37^6/7^ Weeks			Gestational Age of 38^0/7^-38^6/7^ Weeks			Gestational Age of 39^0/7^-39^6/7^ Weeks			Gestational Age of 40^0/7^-40^6/7^ Weeks		
Outcome	Planned Delivery, n=177	Expectant Management, n=4,728	*P*	Planned Delivery, n=392	Expectant Management, n=3,736	*P*	Planned Delivery, n=534	Expectant Management, n=2,434	*P*	Planned Delivery, n=458	Expectant Management, n=1,218	*P*
Perinatal death	0	14 (0.3)	0.47	0	9 (0.2)	0.33	1 (0.2)	6 (0.3)	0.80	1 (0.2)	2 (0.2)	0.82
Stillbirth	0	8 (0.2)	0.58	0	4 (0.1)	0.52	0	3 (0.1)	0.41	0	1 (0.1)	0.54
Neonatal death	0	6 (0.1)	0.64	0	5 (0.1)	0.47	1 (0.2)	3 (0.1)	0.72	1 (0.2)	1 (0.1)	0.47
Adverse neonatal event^a^	3 (1.7)	68 (1.4)	0.78	4 (1.0)	51 (1.4)	0.57	9 (1.7)	31 (1.3)	0.46	3 (0.7)	21 (1.7)	0.10

Note: Data are presented as n (%).

^a^Adverse neonatal event is defined as assisted ventilation >30 minutes, birth injury, seizures, or 5-minute Apgar score ≤3.

When considering only patients with insulin-dependent diabetes (n=1,012), 66 (6.5%) underwent a planned delivery between 37^0/7^ and 37^6/7^ weeks (Table 3). The primary outcome of perinatal death occurred in no patients in the planned delivery group and in 5 (0.5%) of the expectantly managed group (*P*=1.00). Of these 5 perinatal deaths, 3 were stillbirths and 2 were neonatal deaths. The adverse neonatal event occurred in 1 (1.5%) of planned deliveries and 15 (1.6%) of expectantly managed pregnancies (*P*=1.00).

**Table 3.** Outcomes of Planned Delivery vs Expectant Management for Insulin-Dependent Patients (n=1,012)

	Gestational Age of 37^0/7^-37^6/7^ Weeks			Gestational Age of 38^0/7^-38^6/7^ Weeks			Gestational Age of 39^0/7^-39^6/7^ Weeks			Gestational Age of 40^0/7^-40^6/7^ Weeks		
Outcome	Planned Delivery, n=66	Expectant Management, n=946	*P*	Planned Delivery, n=149	Expectant Management, n=642	*P*	Planned Delivery, n=124	Expectant Management, n=363	*P*	Planned Delivery, n=74	Expectant Management, n=184	*P*
Perinatal death	0	5 (0.5)	1.00	0	3 (0.5)	1.00	0	2 (0.6)	1.00	1 (1.4)	0	0.29
Stillbirth	0	3 (0.3)	1.00	0	2 (0.3)	1.00	0	1 (0.3)	1.00	0	0	–
Neonatal death	0	2 (0.2)	1.00	0	1 (0.2)	1.00	0	1 (0.3)	1.00	1 (1.4)	0	0.29
Adverse neonatal event^a^	1 (1.5)	15 (1.6)	1.00	1 (0.7)	9 (1.4)	0.70	0	6 (1.7)	0.35	1 (1.4)	5 (2.7)	0.68

Note: Data are presented as n (%).

^a^Adverse neonatal event is defined as assisted ventilation >30 minutes, birth injury, seizures, or 5-minute Apgar score ≤3.

### 38 Weeks

Of the 4,905 patients overall, 4,128 (84.2%) reached 38 weeks, and of these, 392 (9.5%) underwent planned delivery between 38^0/7^ and 38^6/7^ weeks (Table 1). Compared to women expectantly managed to ≥39 weeks, women undergoing a planned delivery were less likely to be nulliparous (84.9% vs 90.3%, *P*<0.01), more likely to be obese (38.4% vs 31.9%), and less likely to be underweight (1.6% vs 3.8%) or normal weight (34.4% vs 41.0%, *P*<0.01). Women undergoing planned delivery between 38^0/7^ and 38^6/7^ weeks were also more likely to have adequate plus prenatal care (56.2% vs 39.0%, *P*<0.01).

No perinatal deaths occurred in the planned delivery group, while 9 (0.2%) occurred in the expectant management group (*P*=0.33, Table 2). Of these, 4 were stillbirths and 5 were neonatal deaths. The composite adverse neonatal outcome occurred in 4 (1.0%) planned deliveries and in 51 (1.4%) of those expectantly managed (*P*=0.57).

When considering only patients who were insulin dependent (n=791, 19.2% of patients reaching ≥38 weeks), 149 (18.8%) underwent a planned delivery between 38^0/7^ and 38^6/7^ weeks. No perinatal deaths occurred in the planned delivery group while 3 (0.5%) occurred in the expectantly managed group (*P*=1.00, Table 3). Of these 3 perinatal deaths, 2 were stillbirths and 1 was a neonatal death. The adverse neonatal event occurred in 1 (0.7%) infant in the planned delivery group and 9 (1.4%) in the expectantly managed group (*P*=0.70).

### 39 Weeks

Of the 2,968 (60.5%) patients who reached 39.0 weeks, 534 (18.0%) underwent a planned delivery between 39^0/7^ and 39^6/7^ weeks (Table 1). Compared to the women expectantly managed, women undergoing a planned delivery were less likely to be married (18.9% vs 23.5%, *P*=0.02) or smoke (12.2% vs 17.0%, *P*<0.01), more likely to be obese (39.0% vs 31.3%, *P*<0.01), and more likely to have adequate plus prenatal care (51.7% vs 32.2%, *P*<0.01).

One (0.2%) perinatal death occurred in the planned delivery group, and 6 (0.3%) occurred in the expectantly managed group (*P*=0.80, Table 2). The perinatal death in the planned delivery group was a neonatal death; the 6 perinatal deaths in the expectantly managed group consisted of 3 stillbirths and 3 neonatal deaths. The composite adverse neonatal event occurred in 9 (1.7%) infants in the planned delivery group and 31 (1.3%) of the infants in the expectantly managed group (*P*=0.46).

Of the patients reaching 39^0/7^ weeks, 487 (16.4%) were insulin dependent. No perinatal deaths occurred in the planned delivery group, while 2 (0.6%)—1 stillbirth and 1 neonatal death—occurred in the expectantly managed group (*P*=1.00, Table 3). The adverse neonatal event occurred in no infant in the planned delivery group and in 6 (1.7%) of the expectantly managed group (*P*=0.35).

### 40 Weeks

Of the 1,676 women (34.2% of the cohort) who reached 40 weeks, 458 (27.3%) underwent a planned delivery between 40^0/7^ and 40^6/7^ weeks (Table 1). Women undergoing planned delivery were less likely to have Women-Infants-Children nutrition program coverage (24.8% vs 35.7%, *P*<0.01) or be married (19.4% vs 24.6%, *P*=0.03) and more likely to have adequate plus prenatal care (40.2% vs 23.7%, *P*<0.01).

One perinatal death (0.2%) occurred in the planned delivery group compared to 2 (0.2%) in the expectant management group (*P*=0.82, Table 2). The perinatal death in the planned delivery group was a neonatal death; in the expectantly managed group, the perinatal deaths were 1 stillbirth and1 neonatal death. The adverse neonatal composite event occurred in 3 (0.7%) infants in the planned delivery group and in 21 (1.7%) infants in the expectantly managed group (*P*=0.10).

Of the 1,676 women who reached 40 weeks, 258 (15.4%) were insulin dependent. Of these, 74 (28.7%) underwent a planned delivery between 40^0/7^ and 40^6/7^ weeks. One perinatal death (1.4%), which was a neonatal death, occurred in the planned delivery group, and none occurred in the expectant management group (*P*=0.29, Table 3). The composite adverse neonatal event occurred in 1 (1.4%) of the planned delivery group compared to 5 (2.7%) in the expectant management group (*P*=0.68).

### Perinatal Deaths and Adverse Perinatal Outcomes

Overall, the risk of perinatal death at any gestational age examined was low (3/1,000 births or lower), as was the risk of the adverse perinatal outcome (<2%). When only patients who were insulin dependent were included in the analysis, the risk of perinatal death at any gestation remained low at 6 per 1,000 births or fewer.

Details of the stillbirths and neonatal deaths are presented in Tables 4 and 5. None of the mothers with stillbirths was of advanced maternal age (>35 years), and only 1 fetus was small for gestational age. Among the neonatal deaths, 2 babies were born to mothers of advanced maternal age, and 1 baby was small for gestational age. Cause of death was not available for 5 of the 6 cases. Because we used a birth cohort, information on diabetic control beyond insulin use and antenatal testing regimen was also not available.

**Table 4.** Details of Stillbirths

	Stillbirth							
Risk Factor	1	2	3	4	5	6	7	8
Fetal								
Gestational age at delivery, weeks	37	37	37	37	38	39	39	40
Birthweight, g	1,616	2,835	3,005	2,722	3,969	3,175	3,402	4,054
Small for gestational age	Yes	No	No	No	No	No	No	No
Maternal								
Age, years	20	23	31	31	28	25	23	24
Insulin-dependent diabetic	No	Yes	No	No	Yes	Yes	No	No
Smoker	No	No	No	No	Yes	Yes	No	Yes

**Table 5.** Details of Neonatal Deaths

	Neonatal Death					
Risk Factor	1	2	3	4	5	6
Fetal						
Gestational age at delivery, weeks	37	38	39	39	40	40
Birthweight, g	4,000	2,818	3,325	4,054	3,827	2,523
Small for gestational age	No	No	No	No	No	Yes
Maternal						
Age, years	18	27	38	38	28	22
Insulin-dependent diabetic	Yes	No	No	No	Yes	No
Smoker	Yes	Yes	No	No	No	Yes

## DISCUSSION

In this large, birth- and death-certificate linked cohort of pregnancies complicated by insulin-dependent and non–insulin-dependent diabetes, the absolute risk of perinatal death and adverse neonatal outcomes was low at every gestational age examined. However, the low risk of neonatal death and the composite adverse neonatal event between 37 and 39 weeks suggests that delivery as early as 37 weeks may be a reasonable option in pregnancies complicated by diabetes, particularly for patients with insulin-dependent diabetes.

Prior studies of the timing of delivery in pregnancy have focused largely on patients with gestational diabetes and on preventing macrosomia, shoulder dystocia, or cesarean section.^10-13^ Through a PubMed search using the keywords “diabetes mellitus” and “induction of labor,” we identified 2 randomized controlled trials of expectant management vs induction of labor.^11,14^ In one study, Kjos et al randomized 200 insulin-requiring patients with diabetes (gestational or Class B) at 38 weeks’ gestation to either induction within 5 days or expectant management until up to 42 weeks’ gestation. While birthweight was higher and macrosomia was more frequent in the expectantly managed group, no other significant differences between groups were noted.^11^ In the other study, Worda et al randomized 100 women with insulin-treated gestational diabetes to labor induction at 38 weeks or expectant management until 40 weeks.^14^ The risks of large for gestational age and cesarean section were similar between the 2 groups, although neonatal hypoglycemia was increased in the 38-week group. Given the sample sizes, these studies did not have power to examine stillbirths, neonatal deaths, or respiratory morbidity. Additionally, both of these studies randomized to expectant management beyond 39^6/7^ weeks, which is the latest time frame for delivery recommended by the ACOG, SMFM, and NICHD.

Rosenstein et al assessed the mortality risk of delivery compared to expectant management in pregnancies complicated by gestational diabetes using the California Vital Statistics Birth Certificate Data linked to the Death Certificate Data and Fetal Death File.^15^ The authors developed a composite mortality rate to estimate the risk of expectant management at each gestational age, incorporating the stillbirth risk during the week of continuing pregnancy plus the infant mortality risk at the gestational age 1 week hence. They showed that expectant management was associated with a higher risk of mortality than delivery between 39 and 40 weeks. However, this study did not include patients with pregestational diabetes nor did it distinguish between patients with insulin-dependent and non–insulin-dependent diabetes.

One strength of our large, retrospective study is that we were able to distinguish between patients with insulin-dependent and non–insulin-dependent diabetes from the birth certificate data. Although we were unable to determine gestational vs pregestational diabetes, the distinction of requiring or not requiring insulin is in line with recommendations to base timing of delivery on whether or not insulin is used.^3^ Additionally, we examined significant neonatal morbidities that have been associated with elective, early-term deliveries.^6,16^ We elected to examine neonatal morbidity in addition to stillbirth because morbidity (particularly respiratory morbidity) may be a potential consequence of early-term delivery and may occur more frequently than stillbirth or neonatal death.

This study has limitations. By using a birth certificate cohort, we were inherently limited by the quality and quantity of information available. No data were available on the time since diagnosis of diabetes, glycemic control throughout pregnancy, or the use of antenatal testing, all of which may impact the risk of stillbirth and the timing of delivery. In comparisons of birth certificate data to medical records, objective information such as live birth, birth weight, gestational age, and mode of delivery agree with fairly good accuracy.^17,18^ The use of checkbox formats improves the accuracy of data contained in birth certificates,^19,20^ but less objective data, such as medical complications or indication for delivery, tend to be underreported or misclassified. In our study, underreporting may have reduced the sample size of women with diabetes available to analyze. Misclassification bias (ie, classifying a subject as expectant management rather than planned delivery) would have biased the findings to the null. The cohort also spans a long time period (1989 to 2005), which was necessary for sufficient numbers of pregnancies complicated by this relatively rare comorbidity, but changes in prenatal and neonatal care may have impacted the frequency of stillbirth and neonatal death throughout the study. Finally, low numbers of planned deliveries prior to 37 weeks and beyond 41 weeks prevented us from examining these gestational age ranges, although some suggest that late preterm birth may be reasonable in pregnancies complicated by diabetes.^3-5^ The low number of outcomes at each gestational age range prohibited the use of logistic regression to adjust for confounding factors.

## CONCLUSION

In this cohort of pregnancies complicated by diabetes, expectant management beyond early term was associated with an increase in the occurrence of perinatal death without a concomitant decrease in significant neonatal morbidities. Although stillbirth is rare, even in this high-risk cohort, the low risk of neonatal death and significant neonatal morbidities suggests that early-term delivery may be a reasonable option in these high-risk pregnancies. Further well-powered studies that can consider diabetic control, medical comorbidities, perinatal care, and long-term outcomes are needed.

## Notes

This article meets the Accreditation Council for Graduate Medical Education and the American Board of Medical Specialties Maintenance of Certification competencies for Patient Care and Medical Knowledge.

## References

[1] Resnik R, Lockwood C, Moore T, Greene M, Copel J, Silver R, eds. Creasy and Resnik's Maternal-Fetal Medicine: Principles and Practice. 8th ed Elsevier; 2018.

[2] Reddy UM, Laughon SK, Sun L, Troendle J, Willinger M, Zhang J Prepregnancy risk factors for antepartum stillbirth in the United States. Obstet Gynecol. 2010;116(5):1119-1126. doi: 10.1097/AOG.0b013e3181f903f8 20966697 PMC3326407

[3] American College of Obstetricians and Gynecologists. ACOG committee opinion no. 764: medically indicated late-preterm and early-term deliveries. Obstet Gynecol. 2019;133(2):e151-e155. doi: 10.1097/AOG.0000000000003083 30681545

[4] Spong CY, Mercer BM, D'alton M, Kilpatrick S, Blackwell S, Saade G Timing of indicated late-preterm and early-term birth. Obstet Gynecol. 2011;118(2 Pt 1):323-333. doi: 10.1097/AOG.0b013e3182255999 21775849 PMC3160133

[5] American College of Obstetricians and Gynecologists. ACOG committee opinion no. 560: medically indicated late-preterm and early-term deliveries. Obstet Gynecol. 2013;121(4):908-910. doi: 10.1097/01.AOG.0000428648.75548.00 23635709

[6] Tita ATN, Landon MB, Spong CY, et al; Eunice Kennedy Shriver NICHD Maternal-Fetal Medicine Units Network. Timing of elective repeat cesarean delivery at term and neonatal outcomes. N Engl J Med. 2009;360(2):111-120. doi: 10.1056/NEJMoa0803267 PMC2811696 19129525

[7] Wilmink FA, Hukkelhoven CWPM, Lunshof S, Mol BWJ, van der Post JAM, Papatsonis DNM Neonatal outcome following elective cesarean section beyond 37 weeks of gestation: a 7-year retrospective analysis of a national registry. Am J Obstet Gynecol. 2010;202(3):250.e1-250.e8. doi: 10.1016/j.ajog.2010.01.052 20207243

[8] Wingate MS, Alexander GR, Buekens P, Vahratian A Comparison of gestational age classifications: date of last menstrual period vs. clinical estimate. Ann Epidemiol. 2007;17(6):425-430. doi: 10.1016/j.annepidem.2007.01.035 17395481

[9] Bloch JR, Dawley K, Suplee PD Application of the Kessner and Kotelchuck prenatal care adequacy indices in a preterm birth population. Public Health Nurs. 2009;26(5):449-459. doi: 10.1111/j.1525-1446.2009.00803.x 19706128

[10] Lurie S, Insler V, Hagay ZJ Induction of labor at 38 to 39 weeks of gestation reduces the incidence of shoulder dystocia in gestational diabetic patients class A2. Am J Perinatol. 1996;13(5):293-296. doi: 10.1055/s-2007-994344 8863948

[11] Kjos SL, Henry OA, Montoro M, Buchanan TA, Mestman JH Insulin-requiring diabetes in pregnancy: a randomized trial of active induction of labor and expectant management. Am J Obstet Gynecol. 1993;169(3):611-615. doi: 10.1016/0002-9378(93)90631-r 8372870

[12] Witkop CT, Neale D, Wilson LM, Bass EB, Nicholson WK Active compared with expectant delivery management in women with gestational diabetes: a systematic review. Obstet Gynecol. 2009;113(1):206-217. doi: 10.1097/AOG.0b013e31818db36f 19104376

[13] Rayburn WF, Sokkary N, Clokey DE, Moore LE, Curet LB Consequences of routine delivery at 38 weeks for A-2 gestational diabetes. J Matern Fetal Neonatal Med. 2005;18(5):333-337. doi: 10.1080/14767050500274187 16390794

[14] Worda K, Bancher-Todesca D, Husslein P, Worda C, Leipold H Randomized controlled trial of induction at 38 weeks versus 40 weeks gestation on maternal and infant outcomes in women with insulin-controlled gestational diabetes. Wien Klin Wochenschr. 2017;129(17-18):618-624. doi: 10.1007/s00508-017-1172-4 28168363

[15] Rosenstein MG, Cheng YW, Snowden JM, Nicholson JM, Doss AE, Caughey AB The risk of stillbirth and infant death stratified by gestational age in women with gestational diabetes. Am J Obstet Gynecol. 2012;206(4):309.e1-309.e7. doi: 10.1016/j.ajog.2012.01.014 22464068 PMC3403365

[16] Salemi JL, Pathak EB, Salihu HM Infant outcomes after elective early-term delivery compared with expectant management. Obstet Gynecol. 2016;127(4):657-666. doi: 10.1097/AOG.0000000000001331 26959207

[17] Buescher PA, Taylor KP, Davis MH, Bowling JM The quality of the new birth certificate data: a validation study in North Carolina. Am J Public Health. 1993;83(8):1163-1165. doi: 10.2105/ajph.83.8.1163 8342728 PMC1695166

[18] Piper JM, Mitchel EF Jr, Snowden M, Hall C, Adams M, Taylor P Validation of 1989 Tennessee birth certificates using maternal and newborn hospital records. Am J Epidemiol. 1993;137(7):758-768. doi: 10.1093/oxfordjournals.aje.a116736 8484367

[19] Frost F, Starzyk P, George S, McLaughlin JF Birth complication reporting: the effect of birth certificate design. Am J Public Health. 1984;74(5):505-506. doi: 10.2105/ajph.74.5.505 6711731 PMC1651619

[20] Woolbright LA, Harshbarger DS The revised standard certificate of live birth: analysis of medical risk factor data from birth certificates in Alabama, 1988-92. Public Health Rep. 1995;110(1):59-63. 7838945 PMC1382075

